# Carbohydrate antigen 19-9 elevation without evidence of malignant or pancreatobiliary diseases

**DOI:** 10.1038/s41598-020-65720-8

**Published:** 2020-06-01

**Authors:** Sunyoung Kim, Byung Kyu Park, Jeong Hun Seo, Jinyoung Choi, Jong Won Choi, Chun Kyon Lee, Jae Bock Chung, Yongjung Park, Dong Wook Kim

**Affiliations:** 1 Division of Gastroenterology, Department of Internal Medicine, National Health Insurance Service Ilsan Hospital, Goyang, Korea; 2 Department of Laboratory Medicine, National Health Insurance Service Ilsan Hospital, Goyang, Korea; 3 Department of Policy Research Affairs, National Health Insurance Service Ilsan Hospital, Goyang, Korea

**Keywords:** Pancreatic cancer, Tumour biomarkers

## Abstract

Although carbohydrate antigen 19-9 (CA 19-9) may be elevated in benign diseases, elevated CA 19-9 may cause a fear of cancer and unnecessary follow-up studies. Research on how to approach systematically in this case is very limited. The purpose of this study was to analyze the clinical features and the causes of CA 19-9 elevation without evidence of malignant or pancreatobiliary diseases. We retrospectively reviewed the medical records of patients who had CA 19-9 elevation (≥80 U/mL) and were found to be unrelated to cancer after follow-up. After exclusion, 192 patients were included in this study. The median level of CA 19-9 was 136.5 U/mL. The causes of CA 19-9 elevation were determined in 147 (76.6%) patients, and that was unknown in 45 (23.4%). The estimated causative diseases were hepatic diseases in 63 patients, pulmonary diseases in 32, gynecologic diseases in 38, endocrine diseases in 13, and spleen disease in 1. Of 45 patients with unknown cause, 35 had normalization of CA 19-9 and 10 had persistently elevated CA 19-9. In conclusion, CA 19-9 elevation without malignancies or pancreatobiliary diseases should be systematically evaluated and followed up. We suggest an algorithm to investigate the causes and follow up these patients.

## Introduction

Tumor markers are useful for screening and diagnosis of cancer in high-risk patients, evaluating the response to cancer treatment and detecting the recurrence of cancer. As health checkups are becoming widely available, tumor markers are being checked in asymptomatic healthy people for screening purposes.

Among the many tumor markers, carbohydrate antigen 19-9 (CA 19-9) has a relatively high sensitivity and specificity for pancreatic and biliary tract tumors^1,2^. According to previous studies, the sensitivity of CA 19-9 in diagnosing pancreatic cancer ranges between 79% and 95%, and the specificity ranges between 82% and 91%^3,4^. CA 19-9 has its diagnostic value when the patient has symptoms such as weight loss, abdominal pain, and jaundice or when imaging studies indicate a tumor. However, in asymptomatic patients, there is little evidence of its clinical value in screening for cancer^5–8^. In a study conducted with 70,940 asymptomatic individuals, the positive predictive value of CA 19-9 was only 0.9% including ineffectiveness in screening for pancreatic cancer^8^.

CA 19-9 is synthesized in the normal pancreatic parenchyma and biliary tract. It is also produced from the epithelial cells of the gastric, colonic, and uterine mucosa, as well as the salivary glands^9^. From a malignant tumor perspective, CA 19-9 has been reported to be increased in stomach, colorectal, lung, thyroid, as well as biliary and pancreatic cancer^10^. CA 19-9 is often elevated in benign pancreatobiliary diseases such as cholangitis, obstructive jaundice and pancreatitis^11,12^. Additionally, it has been reported that elevated CA 19-9 levels can be found in other non-malignant conditions such as pulmonary and thyroidal diseases, diabetes mellitus, and gynecologic diseases^5,13,14^. In patients with an elevated CA 19-9 level, sufficient examination including an abdominal computed tomography (CT) scan in search of malignant diseases is generally performed first. When no malignant disease is found, a short-term follow-up is usually recommended^5,6^. Although elevated CA 19-9 level in healthy individuals is widely accepted to be unrelated to malignant diseases, patients with elevated CA 19-9 often present to the hospital in the fear of cancer. In these cases, evaluating the exact cause of its elevation is very important in reassuring anxious patients and avoiding unnecessary follow-up studies. However, there are no studies on the systematic approach to benign diseases that cause CA 19-9 elevation, and only a few case reports have been published.

The purpose of this study was to analyze the possible causes of CA 19-9 elevation in non-cancerous patients based on the follow-up studies by reviewing their medical records retrospectively. Furthermore, we suggest a systematic approach for patients with elevated CA 19-9 unrelated to malignant or pancreatobiliary diseases.

## Results

A total of 6,899 cases were found with a CA 19-9 level ≥80 U/ml. After reviewing the medical records, patients with malignancies, benign pancreatobiliary diseases, or patients with no follow-up medical records were excluded. Finally, 192 patients were selected for this study.

The average age of the patients was 53.33 ± 16.81 years with 77 (40.1%) men and 115 (59.9%) women. The median CA 19-9 of the selected patients was 136.5 (range: 80.2-more than 1,000) U/mL. The following diagnostic imaging tests were performed to determine the cause of serum CA 19-9 elevation: Chest X-ray in 191 patients, chest CT in 32, abdominal CT in 192, magnetic resonance imaging in 6, positron emission tomography in 4, abdominal ultrasound in 78, gynecologic ultrasound in 45, esophagogastroduodenoscopy in 76, and colonoscopy in 24 patients. The mean follow-up period for subsequent cancer occurrence was 6.76 ± 3.18 years. None of subjects were subsequently diagnosed with malignant diseases within 2 years of CA 19-9 elevation. After 2 years of CA 19-9 elevation, subsequent cancers developed in 4 patients, which included rectal cancer, stomach cancer, lung cancer, and hepatocellular carcinoma. Each cancer was diagnosed 2.9-10.9 years after the CA 19-9 elevation.

The estimated causes of CA 19-9 elevation were found in 147 (76.6%) patients; no possible explanation could be found in 45 (23.4%) patients. The estimated diseases that caused CA 19-9 elevation were hepatic in 63 patients, pulmonary in 32, gynecologic in 38, endocrine in 13, and spleen disease in 1 (Table 1).

**Table 1:** Category of etiologic diseases for elevated CA 19-9 without evidence of malignant or pancreatobiliary diseases.

Etiologic diseases	No. of subjects
Hepatic diseases	63 (32.8%)
Pulmonary diseases	32 (16.7%)
Gynecologic diseases	38 (19.8%)
Endocrine diseases	13 (6.8%)
Spleen cyst	1 (0.5%)
Unknown cause	45 (23.4%)

### Hepatic diseases

CA 19-9 elevation was associated with hepatic disease in 63 cases. The estimated causative diseases are as follows: 16 had alcoholic liver cirrhosis, 13 had alcoholic hepatitis, 12 had drug induced hepatitis, nine had acute viral hepatitis, nine had chronic hepatitis B, three had autoimmune hepatitis, and one had a liver cyst (Table 2). Of 9 patients with acute hepatitis, three had acute hepatitis A, three had acute hepatitis B, and 3 had non-A non-B acute hepatitis. CA 19-9 increased up to 705.8 U/mL in these patients. Of the 3 cases of autoimmune hepatitis, 2 were confirmed by liver biopsy. The other one was clinically diagnosed with a positive anti-nuclear antibody and treated with a corticosteroid for 4 years. The median level of CA 19-9 level was relatively high for autoimmune hepatitis at 403.0 U/mL. Total bilirubin, aspartate aminotransferase (AST), and alanine aminotransferase (ALT) were markedly increased with liver injury in most cases.

**Table 2:** Elevation of CA 19-9 in benign hepatic diseases. L/C, liver cirrhosis; T-bil, total bilirubine; AST, aspartate transferase; ALT, alanine transferase.

Diagnosis	No. of subjects	CA 19-9 (U/mL) Median (Range)	Median of T- bil (mg/dL)	Median of AST (U/mL)	Median of ALT (U/mL)
Alcoholic L/C	16	129.9 (108.2-512.0)	4.1	135.0	59.0
Alcoholic hepatitis	13	150.7 (80.6-366.8)	4.7	209.0	132.0
Drug-induced hepatitis	12	160.8 (93.9-688.4)	9.7	642.0	534.5
Acute hepatitis	9	129.0 (81.8-705.8)	7.7	746.0	811.0
Chronic hepatitis B	9	122.6 (105.0-493.0)	1.6	198.5	312.0
Autoimmune hepatitis	3	403.0 (81.2-645.0)	7.1	912.0	668.0
Liver cyst	1	460.2			

**Figure 1:**
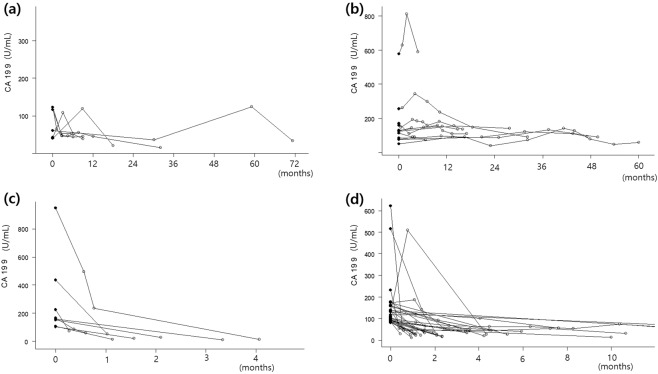
Spider blot of changes of CA 19-9 in patients with follow-up CA 19-9. (**a**) Patients with chronic hepatitis B; (**b**) Patients with bronchiectasis; (**c)** Patients undergoing surgery for endometriosis; (**d**) Patients in whom the cause of CA 19-9 elevation was unclear and CA 19-9 normalized after follow-up (●: initial CA 19-9, ○: follow-up CA 19-9).

For most of the hepatic disease cases, CA 19-9 levels had been checked to rule out malignant disease when the patient had abnormal liver function test results or clinical signs related to worsened liver function. CA 19-9 levels had been normalized or markedly decreased in most patients after improvement of liver function. In patients with chronic hepatitis B, CA 19-9 increased or decreased with changes in AST and ALT but did not increase steadily (Fig. 1a). In one case with a liver cyst, the cyst was measured to be 10 cm in size with peripheral biliary tract dilatation and the CA 19-9 level was elevated up to 460.2 U/mL in this patient and remained consistently elevated without evidence of malignancy during the follow-up.

### Pulmonary diseases

Pulmonary disease was implicated with CA 19-9 elevation in 32 cases. There were 14 cases of bronchiectasis, eight of interstitial lung disease, five of pulmonary tuberculosis, two of lung abscess, two of pneumonia, and one of pulmonary sequestration (Table 3). The median CA 19-9 was 137.7 U/mL in those with bronchiectasis, and 225.0 U/mL in those with interstitial lung disease. In one case of bronchiectasis, the CA 19-9 was elevated to as high as 810.2 U/mL.

**Table 3:** Elevation of CA 19-9 in benign pulmonary, gynecologic, endocrine, spleen diseases and unknown cause. DM, diabetes mellitus.

Diagnosis	No. of subjects	CA 19-9 (U/mL)	
		Median	Range
**Pulmonary diseases**			
Bronchiectasis	14	137.7	105.6–810.2
Interstitial lung disease	8	225.0	114.0–298.4
Pulmonary tuberculosis	5	185.5	102.6–229.3
Lung abscess	2	209.9	165.7–254.0
Pneumonia	2	149.0	130.0–168.0
Pulmonary sequestration	1	>1,000	
**Gynecologic disease**			
Cystic teratoma	19	150.9	82.6–452.9
Endometriosis	18	135.8	93.7–948.2
Ovarian abscess	1	570.0	
**Endocrine diseases**			
Uncontrolled DM	10	106.5	80.3–186.0
Hypothyroidism	3	96.0	89.9–147.1
**Spleen disease**			
Spleen cyst sclerotherapy	1	218.6	
Unknown cause	45	119.3	80.2–590.5

Interestingly, in one case of pulmonary sequestration, the CA 19-9 level continuously increased up to more than 1,000 U/mL without developing any malignancy during 2 years of follow-up. In chronic lung disease, the CA 19-9 level remained consistently elevated with a slight increase or decrease, but never normalized. The changes of CA 19-9 in bronchiectasis patients are shown in Fig. 1b. However, in acute pulmonary diseases such as pneumonia or lung abscess, the CA 19-9 level normalized or decreased after treatment.

### Gynecologic diseases

There were 38 cases of elevated CA 19-9 with gynecological disease. Among them, 19 had cystic teratoma, 18 had endometriosis, and one had an ovarian abscess (Table 3). The median value of CA 19-9 was 150.9 U/ml for cystic teratoma and 135.8 U/mL for endometriosis. One case of cystic teratoma had a CA 19-9 level of 452.9 U/mL and one case of endometriosis had a CA 19-9 level of 948.2 U/mL.

The CA 19-9 level normalized in patients who had undergone surgery to remove the endometriosis or cystic teratoma. The normalization of CA 19-9 after surgery for endometriosis was shown in Fig. 1c. The CA 19-9 level was elevated to as high as 570.0 U/mL in one patient with an ovarian abscess, which normalized after treatment.

### Endocrine diseases

**Figure 2:**
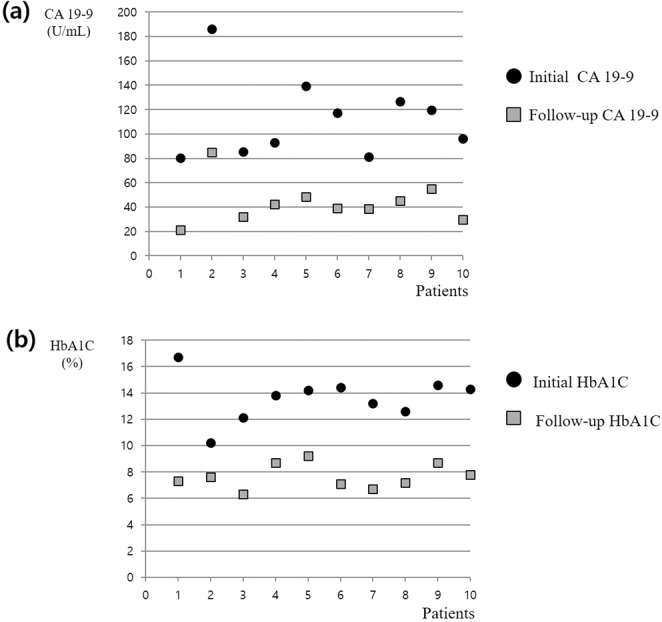
Changes of CA 19-9 and hemoglobin A1C in subjects with uncontrolled diabetes mellitus after sugar control. (**a**) Initial CA 19-9 was elevated more than 80 U/ml in all subjects. After control of the blood glucose, follow-up CA 19-9 was markedly decreased in all subjects; (**b**) Hemoglobin A1C was decreased after control of the blood glucose in all subjects. HbA1C, hemoglobin A1C.

There were 13 cases of CA 19-9 elevation due to endocrine disease (Table 3). The median value of CA 19-9 was 106.5 U/mL for 10 cases of uncontrolled diabetes mellitus. The initial average level of hemoglobin A1C (HbA1c) in these patients was 13.61% (10.2–16.7). After controlling the blood glucose, it decreased to 7.66% (6.3–9.2) with CA 19-9 also decreasing dramatically to the mean value of 43.7 U/mL in all patients (Fig. 2). There were three cases of hypothyroidism with a median CA 19-9 value of 96.0 U/mL.

### Spleen disease

There was one case of elevated CA 19-9 in post-sclerotherapy of a splenic cyst (Table 3). A 23-year-old woman had a 6.5 cm sized symptomatic spleen cyst, and the pre-procedural serum CA 19-9 was mildly elevated to 46.69 U/mL. Six days later, the patient received sclerotherapy with 95% alcohol for spleen cyst, and the day after the procedure, the serum CA 19-9 was markedly elevated to 218.6 U/mL. After 1 year, the size of the spleen cyst was markedly decreased to 1.5 cm in the follow-up CT, and she had no cancer occurrence over the next 8 years.

### Unknown cause

The cause of CA 19-9 elevation higher than 80 U/mL in 45 cases remained unknown despite investigation of blood test results, imaging studies, and follow-up studies. The median value of CA 19-9 was 119.3 U/mL. Among these cases, 35 had normalized levels and only 10 continued to have elevated levels at the follow-ups. The time required for CA 19-9 normalization was less than 1 month in 10 patients, 1~2 months in 10, 2~3 months in five, 3~4 months in five, 4~6 months in three, and 1~2 years in two patients (Fig. 1d). In 71.4% (25/35) of cases, CA 19-9 normalized within 3 months.

## Discussion

CA 19-9 is a tumor marker that is frequently checked in healthy individuals when screening for various cancers including pancreatic cancer^5–8^. In addition to malignancies, it is known that CA 19-9 may also increase in benign pancreatobiliary, hepatic and pulmonary diseases, thyroiditis, diabetes mellitus, and autoimmune diseases. Therefore, we should be cautious in interpreting the significance of CA 19-9 elevation^5,13^.

The reason for CA 19-9 elevation in benign disease can be explained by several mechanisms. First of all, inflammation and proliferation of non-tumorous tissue, such as in pancreatitis, cholangitis, bronchiectasis, idiopathic pulmonary fibrosis, ovarian cyst, and endometriosis, can elevate CA 19-9. CA 19-9 also increases as a result of obstructive jaundice and bronchitis as CA 19-9 excretion is blocked in these diseases. The last mechanism is when metabolic malfunction is present such as in hepatitis, diabetes mellitus, and chronic glomerulonephritis^14^.

In this study, the characteristics of CA 19-9 elevation in benign disease were analyzed. In clinical practice, physicians often encounter patients with slightly elevated CA 19-9. However, CA 19-9 level that is higher than twice the normal range (≥80 U/mL) is relatively uncommon. In these cases, careful evaluation should be done to rule out malignancies. Most of the previous studies included patients with CA 19-9 > 37 U/mL, however to be more specific, we limited the study population to patients with CA 19-9 ≥ 80 U/mL. Benign pancreatobiliary diseases are a well-known cause of CA 19-9 elevation^11,12^. Especially in the case of acute cholangitis, CA 19-9 may be elevated higher than 1,000 U/mL. In most of the cases, the level usually returns to normal when the obstruction is properly drained. For this reason, both patients with malignancies and benign pancreatobiliary diseases were excluded from this study.

We retrospectively reviewed the blood test results and imaging studies of 192 selected patients and found the estimated causes of CA 19-9 elevation in 147 (76.6%) patients. We also analyzed to what extent each causative disease contributed to CA 19-9 elevation. The association between benign causative disease and CA 19-9 elevation was also defined under a certain principle, and it is unlikely that two situations are coincidental coexistence.

In hepatic disease, previous studies reported that the CA 19-9 level increases with the progression of hepatic fibrosis and viral hepatitis^15,16^. From our study, the most common cause of CA 19-9 elevation was hepatic disease with 63 cases. Bilirubin, AST, and ALT were increased in most of the hepatic diseases and the mean CA 19-9 was high to 164.9 to 442.5 U/mL. Immunoreactivity of CA 19-9 was observed in the bile ductules and interlobular bile ducts in patients with chronic hepatitis and liver cirrhosis^17^. Also, immunoreactivity of CA 19-9 was high in hepatic inflammatory areas such as bile ductule cells and hepatocytes in metaplasia^16^. In autoimmune hepatitis, proliferated bile ductule cells were positive for CA 19-9 and proliferating cell nuclear antigens^18^. Therefore, CA 19-9 may be produced in these cells, leading to serum CA 19-9 elevation.

There were 32 cases of pulmonary diseases identified in our study. Bronchiectasis and interstitial lung disease were the most common causes of CA 19-9 elevation; the median CA 19-9 levels were 137.7 and 225.0 U/mL, respectively. For chronic lung disease, follow-up study of the CA 19-9 level showed a wax and wane pattern but a significant increase or normalization was not found. According to Kodama *et al*., CA 19-9 is elevated in 38.9% of chronic lung diseases such as idiopathic interstitial lung disease, pulmonary fibrosis associated with connective tissue, disseminated bronchiolitis and bronchiectasis^19^. CA 19-9 is synthesized and secreted by normal bronchial epithelial cells, and serum CA 19-9 is elevated by the extravasation of mucus glycoprotein hypersecreted from bronchiolar epithelial cells^20,21^. The exact reason for CA 19-9 elevation in chronic lung disease is still not verified, but it is presumed to be related to the inflammatory process of lung tissue^19,22^. CA 19-9 was suggested as a biomarker of disease progression for idiopathic pulmonary fibrosis^23^.

Several previous epidemiological studies have shown that diabetic patients are susceptible to pancreatic cancer^24–26^. It has already been shown that CA 19-9 and HbA1c values are mutually correlated with each other and that CA 19-9 increases in proportion to blood sugar levels^27,28^. Therefore, CA 19-9 elevation in diabetic patients should be carefully analyzed^29^. In particular, CA 19-9 elevation in diabetic patients may lead to more fear of pancreas cancer. Uncontrolled diabetes mellitus was responsible for CA 19-9 elevation in 10 patients in this study. All of the patients’ HbA1c levels were markedly elevated. After careful regulation of blood sugar levels, both the HbA1c and CA 19-9 level decreased. Therefore, for diabetic patients with CA 19-9 elevation, an imaging study should be performed to rule out pancreatic cancer. If no abnormality is observed on imaging, we recommend that HbA1c should be checked first because uncontrolled diabetes mellitus may be the cause. If the HbA1c is elevated, the blood glucose should be strictly controlled with follow-up measurements of CA19-9 and HbA1c together. If CA 19-9 decreases after intensive blood sugar control, we could speculate that CA 19-9 elevation was due to hyperglycemia^30,31^.

In gynecological disease, CA 19-9 may increase in conjunction with CA 125 in cystic teratomas^32,33^. As for endometriosis, CA 19-9 is elevated in proportion to the severity of the disease^34^. Thus, when a young female patient presents with CA 19-9 elevation, if there is no malignant disease, we recommend checking for gynecologic diseases as the first step.

A giant spleen cyst has been reported to be associated with serum CA 19-9 elevation^35–38^, but the mechanism remains unclear. The inner epithelial cells of the spleen cyst were immunohistochemically positive for CA 19-9 in some cases, so it can be presumed that CA19-9 can be secreted from these cells^37,38^. When the spleen cyst is ruptured, serum CA 19-9 may be temporarily highly elevated^36^. In a case from this study, serum CA 19-9 was elevated to 218 U/mL the day after sclerotherapy. It is thought that the CA 19-9 secretion temporarily increased due to damage to the epithelial cells by sclerotherapy.

Previous studies on the association between CA 19-9 elevation and benign diseases have been conducted mostly on asymptomatic individuals who had been diagnosed as being healthy upon regular health checkups^5–8,22^. This study included not only the heathy subjects from regular health checkups but also symptomatic patients whose CA 19-9 level was evaluated to rule out malignancies. We should be careful when interpreting CA 19-9 elevation, even in symptomatic patients, since CA 19-9 can also be elevated in benign disease. If malignant disease is not found after an initial work-up, possible benign conditions should be evaluated. Confirmation of the exact benign cause of CA 19-9 elevation has a very important clinical value in that anxiety and unnecessary follow-up tests can be eliminated in patients. In asymptomatic individuals, CA 19-9 has little clinical value^5–8^. Therefore, to reduce unnecessary investigations or anxiety about CA 19-9 elevation, it is important point is to conduct the CA 19-9 test only when clinically indicated, not when a patient is asymptomatic.

**Figure 3:**
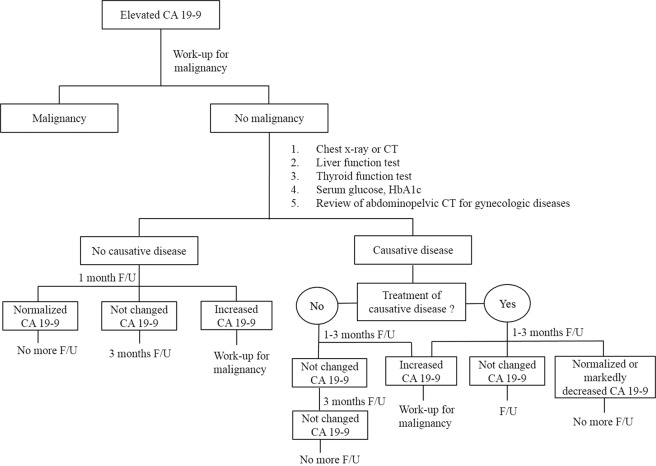
An algorism of systematic approach to CA 19-9 elevation without evidence of malignant or pancraetobiliary diseases. F/U, follow-up; CT, computed tomography; HbA1C, hemoglobin A1C.

This study indicates the benign diseases most frequently causing CA 19-9 elevation and the range of elevated CA 19-9 level in each disease. Based on this result, we suggest performing the following diagnostic strategy when investigating the cause of CA 19-9 elevation without evidence of malignant or pancreatobiliary diseases: (1) chest X-ray or chest CT, (2) serum glucose and HbA1c, (3) liver function test, (4) thyroid function test, and (5) review of an abdominopelvic CT for gynecologic diseases. The diagnosis can be confirmed clearly if changes in CA 19-9 match the course of the suspected benign disease during a follow-up period of 1-3 months. If CA 19-9 is normalized after treatment of the causative disease, additional follow-up is not necessary. CA19-9 will continue to maintain a constant elevated level without major changes if it is not feasible to treat the causative disease such as bronchiectasis. Therefore, no further follow-up is necessary if CA 19-9 does not increase in the initial two follow-ups (Fig. 3).

Even after searching for an appropriate reason for CA 19-9 elevation, there remained a few cases with no possible cause. Tong *et al*. reported that 218 (42.8%) of the 502 subjects with elevated CA 19-9 in a health screen had no precise cause. Of these, 58 (26.6%) had consistently elevated CA 19-9^6^. Ventrucci *et al*. reported on 10 patients with constant CA 19-9 elevation without any malignancies, their CA 19-9 ranged from 112 to 1,338 U/mL^13^. In our study, there were 45 cases in which the cause of CA 19-9 elevation remained unknown after investigation. In 10 patients among them, the CA 19-9 remained consistently high without causative disease during the follow-up period. It is well known that the Lewis (*FUT3*) and Secretor (*FUT2*) genes regulate CA 19-9 secretion^39–41^. Lewis inactivity rarely leads to secretion of CA 19-9, and Secretor inactivity can increase CA 19-9 secretion. The patients were divided into three subgroups based on their genotypes: Lewis(−), Secretor(−)/Lewis(+), and Secretor(+)/Lewis(+) group^39^. The serum CA 19-9 level could be high in Secretor(−)/Lewis(+) group. It should be taken into account that the serum CA 19-9 level is affected by genotypes when there is CA 19-9 elevation without a causative disease.

Most benign diseases are characterized by a decrease or constant value of CA 19-9 after follow-up. On the other hand, CA 19-9 in malignant diseases can be markedly elevated even after a short period of follow-up^6^. Therefore, when the cause of CA 19-9 elevation is unknown even after a systematic screening, these patients should undergo a follow-up study after 1 month. If CA 19-9 remains consistently elevated with no change, we recommend rechecking CA 19-9 again after 3 months. If CA 19-9 level markedly decreased or normalized, a consecutive follow-up study of CA 19-9 has no clinical benefit even if the exact cause of elevation was not explained. If CA 19-9 continuously increases, the need for further investigation to rule out malignancies is of paramount importance (Fig. 3).

A limitation of this study is that it was a retrospective study and the reasons for the CA 19-9 test varied by subjects, so the causes of the CA 19-9 elevation in benign diseases could not represent the frequency of the causal diseases in the actual clinical situation. The second limitation is that it was impossible to eliminate the possibility of the laboratory errors. However, our hospital has quality management for CA 19-9 test. In addition, the laboratory physician (Y. Park) rechecked the possibility of laboratory error in subjects whose CA 19-9 elevated without causes and returned to normal within one month. No evidence of test error was found. The third limitation is that association between benign disease and CA 19-9 elevation was presumed but lacking of definite evidence. Only reproducibility of CA 19-9 elevation by benign disease will be definite evidence. However, it is difficult in a clinical setting and not possible in this retrospective study. Nevertheless, we think that the principle of determining causative diseases in this study is practical and persuasive.

In conclusion, a systematic approach should be performed to identify the benign disease causing CA 19-9 elevation in patients without evidence of malignant or pancreatobiliary diseases. We suggest an algorithm to investigate the causes and follow up these patients. Parameters and tests to be examined and reviewed are chest x-ray or CT, review of abdominal CT for gynecologic disease, liver function, blood sugar and HbA1C, and thyroid function. Through this process, patients can avoid the fear of hidden malignant diseases and can be spared from an unnecessary follow-up study.

## Methods

### Patients

From March 1, 2005 to December 31, 2014, patients with a serum CA 19-9 level higher than twice the normal range (≥80 U/mL) were selected from those who presented to the National Health Insurance Service Ilsan Hospital. Among the selected group, patients who had either malignant diseases such as pancreatic, gallbladder, biliary tract, colorectal, gastric, lung, ovarian, cervix, and endometrial cancer, or a benign pancreatobiliary disease such as acute pancreatitis, chronic pancreatitis, cholangitis, and cholecystitis were excluded. Patients who had not undergone abdominal CT or who had no medical record of further follow-ups were also excluded. The medical records of enrolled patients were analyzed retrospectively based on sex, age, symptoms, chest x-ray, chest CT, abdominal CT, abdominal ultrasound, fasting plasma glucose, HbA1c, liver function test, kidney function test, complete blood test, rheumatoid factor, thyroid function test, neck ultrasound, gynecologic ultrasound, esophagogastroduodenoscopy, colonoscopy, the initial CA 19-9 and the follow-up CA 19-9. We defined that CA 19-9 elevation was not associated with malignant disease when the CA 19-9 level was normalized at the follow-up or if there was no occurrence of malignancy during the follow-up period of 2 years or more. By December 31, 2017, the medical records were reviewed for confirmation of no subsequent occurrence of malignant diseases within 2 years after CA 19-9 elevation. In addition, to exclude the possibility of cancer diagnosed by other hospitals, V193, a specific code of cancer diagnosis in Korea, was checked in each patient’s health insurance eligibility information. Since 2005, the National Health Insurance Service has included a policy that reimburses the payment of cancer patients, who are identified with the specialized claim code of V193.

### Association between CA 19-9 elevation and benign diseases

No relationship between CA 19-9 elevation and malignant disease was defined as follows. First, CA 19-9 was normalized at the follow-up. Second, when CA 19-9 continued to be elevated at the follow-up, there was no occurrence of a malignant disease for 2 years on the medical records. We assumed CA 19-9 elevation was due to a benign disease in the following cases. (1) The patient had a benign disease related to CA 19-9 elevation from previous reports and did not develop malignancies in later follow-ups. (2) The CA 19-9 level normalized after treatment of the diagnosed benign disease. (3) If the diagnosed benign disease was untreatable, the CA 19-9 level should correspond with the wax and wane pattern of the causal disease. (4) If the relationship between the disease and CA 19-9 level was not previously reported, the association was assumed to be relevant if CA 19-9 increased with the disease progression and decreased with the treatment of the disease.

### Statistical analysis

Statistical data were expressed as median. For statistical analyses, SPSS v23 (SPSS Inc., Chicago, IL, USA) was used. R software version 3.5.1 using the “lattice” package for creating the spider blot, and 2-side p value <0.05 was considered statistically significant.

### Ethics

This study design was reviewed and approved by the Institutional Review Board of National Health Insurance Service Ilsan Hospital (NHIMC 2015-04-024). Because the current study was performed with a retrospective design, informed consent was waived by the board. All experiments and methods were conducted in accordance with the Declaration of Helsinki.

## Data Availability

The data that support the findings of this study are available from the corresponding author [B. K. Park] upon reasonable request.
